# Two variables functionals and inequalities related to measurable operators

**DOI:** 10.1186/s13660-017-1583-9

**Published:** 2017-12-12

**Authors:** Jingjing Shao

**Affiliations:** College of Mathematics and Statistics Science, Ludong University, Yantai, 264000 China

**Keywords:** 46L52, 47A63, von Neumann algebra, *τ*-measurable operators, interpolated inequality, Young inequality, Heinz inequality

## Abstract

In this paper, we introduce two variables norm functionals of *τ*-measurable operators and establish their joint log-convexity. Applications of this log-convexity will include interpolated Young, Heinz and Trace inequalities related to *τ*-measurable operators. Additionally, interpolated versions and their monotonicity will be presented as well.

## Introduction

Let $M_{n}$ be the space of $n\times n$ complex matrices and $M_{n}^{+}$ be the class of $M_{n}$ consisting of positive semi-definite matrices. Recall that a norm $|\!|\!|\cdot |\!|\!|$ on $M_{n}$ is symmetric, whenever $|\!|\!|UAV|\!|\!|=|\!|\!|A|\!|\!|$ for all $A\in M_{n}$ and all unitary $U, V\in M_{n}$.

In [1], Bourin and Lee introduced a two variables functional and established the following log-convexity theorem: Let $A, B\in M_{n} ^{+}$ and $z\in M_{n}$. Then, for all symmetric norms and $\alpha >0$, the function

1.1 $$\begin{aligned} f(p,t)=\big|\!\big|\!\big|\bigl\vert A^{\frac{t}{p}}zB^{\frac{t}{p}} \bigr\vert ^{\alpha p}\big|\!\big|\!\big| \end{aligned}$$

is joint log-convex on $(0, \infty)\times (0, \infty)$. By an application of this log-convexity theorem, Bourin and Lee improved many remarkable matrix inequalities, most of which were related to Araki type inequalities. Moreover, fixing one variable in the functional in (1.1) yielded the following log-majorization:

1.2 $$\begin{aligned} \bigl(AZ^{*}BZA \bigr)^{p}\prec_{w \log} A^{p}Z^{*}B^{p}ZA^{p}. \end{aligned}$$

Fixing the other variable in (1.1) entailed a Hölder inequality due to Kosaki (see Theorem 3 of [2]) or Bourin and Lee (see Corollary 3.3 of [1]). Additionally, several matrix versions of an inequality of Littlewood related to the Hölder inequality were obtained.

Interpolated Young and Heinz inequalities for numbers and matrices were first given in [3]. In [4], Sababheh presented the following log-convexity result for symmetric norms: Let $A, B\in M_{n}^{+}$ and $X\in M_{n}$. Then the function $f(p,q)=|\!|\!|A^{p}XB^{q}|\!|\!|$ is log-convex on $(0,\infty)\times (0,\infty)$. With this result, the interpolated Young and Heinz inequalities for matrices as well as related refined inequalities were established. Among other things, the following monotonicity results that the interpolated inequalities obey were proved: Let $A, B\in M_{n}^{+}$, $X\in M_{n}$ and $p\geq q>0$. Then the functions

$$f(r)=\frac{p-q+r}{p-q+2r}\big|\!\big|\!\big|A^{p+r}XB^{q-r}\big|\!\big|\!\big|+ \frac{r}{p-q+2r}\big|\!\big|\!\big|A^{q-r}XB^{p+r}\big|\!\big|\!\big|$$

and

$$f(r)=\big|\!\big|\!\big|A^{p+r}XB^{q-r}\big|\!\big|\!\big|^{\frac{p-q+r}{p-q+2r}} \big|\!\big|\!\big|A^{q-r}XB^{p+r}\big|\!\big|\!\big|^{ \frac{r}{p-q+2r}} $$

are increasing on $[0,q]$. Note that this result in turn guaranteed the interpolated Young and Heinz inequalities, also the related refined inequalities (see [3]).

Let $\mathcal{M}$ be a semi-finite von Neumann algebra acting on the Hilbert space $\mathcal{H}$ with a normal semi-finite faithful trace *τ* and $E(\mathcal{M})$ be a noncommutative symmetric quasi-Banach space. Via the notion of generalized singular value studied by Fack and Kosaki [5], the paper aims to derive some trace inequalities as well as some interpolated Young inequalities for measurable operators, also the related refined inequalities. These inequalities are given in Section 3. Moreover, the hidden monotonicity behavior these inequalities obey is discussed. In Section 4, some variations of Heinz inequalities and the interpolated Heinz inequalities as well as the related refined inequalities are established. Similarly, the monotonicity these inequalities obey is also clarified. Moreover, it is necessary to mention that the operator generalization of the Hölder inequality stated forward was given by Bekjan and Ospanov (see [6]).

The main results in this work are the log-convexity theorems of a two variables functional of measurable operators (see Lemma 3.10 and Theorem 3.11), which are generalizations of the results of Bourin and Lee [1].

## Preliminaries

In this section, we collect some of the basic facts and notation that will be used in the paper. And we recall some classical notations.

A positive function *f* is called log-convex on $(0, \infty)$ if, for all $s, t\in (0, \infty)$ and $\alpha \in [0, 1]$,

$$f \bigl(\alpha t +(1-\alpha)s \bigr)\leq f(t)^{\alpha }f(s)^{1-\alpha }. $$

A positive function *g* is called joint log-convex on $(0, \infty) \times (0, \infty)$ if, for all $t_{i}, s_{i}\in (0, \infty)$, $i=1, 2$ and $\alpha \in [0, 1]$,

$$f \bigl(\alpha (t_{1}, s_{1}) +(1-\alpha) (t_{2}, s_{2}) \bigr)\leq f(t_{1}, s _{1})^{\alpha }f(t_{2}, s_{2})^{1-\alpha }. $$

Unless stated otherwise, throughout the paper $\mathcal{M}$ will always denote a semi-finite von Neumann algebra acting on the Hilbert space $\mathcal{H}$ with a normal semi-finite faithful trace *τ*. We refer to [7, 8] for standard facts concerning von Neumann algebras and [9, 10] for noncommutative integration. The identity in $\mathcal{M}$ is denoted by 1, and let $\mathcal{P}$ denote the projection lattice of $\mathcal{M}$. A closed densely defined linear operator *x* in $\mathcal{H}$ with domain $D(x)\subseteq \mathcal{H}$ is said to be affiliated with $\mathcal{M}$ if $u^{*}xu=x$ for all unitary operators *u* which belong to the commutant $\mathcal{M^{ \prime }}$ of $\mathcal{M}$. If *x* is affiliated with $\mathcal{M}$, we define its distribution function by $\lambda_{s}(x)=\tau (e^{ \bot }_{s}(\vert x\vert ))$, and *x* will be called *τ*-measurable if and only if $\lambda_{s}(x)<\infty $ for some $s>0$, where $e^{\bot }_{s}(\vert x\vert )=e _{(s, \infty)}(\vert x\vert )$ is the spectral projection of $\vert x\vert $ associated with the interval $(s, \infty)$. Moreover, if $\mathcal{M}$ is a finite von Neumann algebra and *x* is affiliated with $\mathcal{M}$, then *x* is automatically *τ*-measurable. The decreasing rearrangement of *x* is defined by $\mu_{t}(x)=\inf \{s>0: \lambda_{s}(x)\leq t\}$, $t>0$. We will denote simply by $\lambda (x)$ and $\mu (x)$ the functions $t\rightarrow \lambda_{t}(x)$ and $t\rightarrow \mu_{t}(x)$, respectively. See [5] for basic properties and detailed information on decreasing rearrangement of *x*.

The set of all *τ*-measurable operators will be denoted by $L_{0}(\mathcal{M},\tau)$, or simply by $L_{0}(\mathcal{M})$. The set $L_{0}(\mathcal{M})$ is a ∗-algebra with sum and product being the respective closures of the algebraic sum and product. The measure topology in $L_{0}(\mathcal{M})$ is the vector space topology defined via the neighborhood base $\{N(\varepsilon, \delta): \varepsilon, \delta >0\}$, where $N(\varepsilon, \delta)=\{x\in L_{0}( \mathcal{M}): \tau (e_{(\varepsilon,\infty)}(\vert x\vert ))\leq \delta \}$ and $e_{(\varepsilon,\infty)}(\vert x\vert )$ is the spectral projection of $\vert x\vert $ associated with the interval $(\varepsilon, \infty)$. With respect to the measure topology, $L_{0}(\mathcal{M})$ is a complete topological ∗-algebra. As usual, we denote by $\Vert \cdot \Vert $ ($=\Vert \cdot \Vert _{ \infty }$) the usual operator norm.

Let *E* be a symmetric Banach space on $(0,\infty)$. We define

$$E(\mathcal{M})= \bigl\{ x\in L_{0}(\mathcal{M}): \mu (x)\in E \bigr\} \quad \mbox{and}\quad \Vert x\Vert _{E(\mathcal{M})}=\bigl\Vert \mu (x)\bigr\Vert _{E}. $$

Then $(E(\mathcal{M}), \Vert \cdot \Vert _{E(\mathcal{M})})$ is a noncommutative symmetric Banach space [11]. A special case of this construction is the noncommutative spaces $L_{p}(\mathcal{M})$, $0< p<\infty $, and the norm $\Vert \cdot \Vert _{p}$ is the corresponding norm on $L_{p}( \mathcal{M})$. As well, $L_{\infty }(\mathcal{M})=\mathcal{M}$. For $0< r<\infty $, we define

$$E(\mathcal{M})^{(r)}= \bigl\{ x\in L_{0}(\mathcal{M}): \vert x\vert ^{r}\in E( \mathcal{M}) \bigr\} \quad \mbox{and}\quad \Vert x \Vert _{E(\mathcal{M})^{(r)}}=\bigl\Vert \vert x\vert ^{r}\bigr\Vert _{E(\mathcal{M})}^{\frac{1}{r}}. $$

It was shown in Proposition 3.1 of [12] that, if *E* is a symmetric (quasi) Banach space, then so is $E(\mathcal{M})^{(r)}$. Moreover, $E^{(r)}(\mathcal{M})=E(\mathcal{M})^{(r)}$, where $E^{(r)}(\mathcal{M})=\{x\in L_{0}(\mathcal{M}): \mu (x)\in E^{(r)}\}$ and $\Vert x\Vert _{E^{(r)}(\mathcal{M})}=\Vert \mu (x)\Vert _{E^{(r)}}$. Let $0< r_{0}, r_{1}, r<\infty $ with $\frac{1}{r}=\frac{1}{r_{0}}+\frac{1}{r _{1}}$. Then the Hölder inequality on $E(\mathcal{M})^{(r)}$ is

2.1 $$\begin{aligned} \Vert xy\Vert _{E(\mathcal{M})^{(r)}}\leq \Vert x\Vert _{E(\mathcal{M})^{(r_{0})}}\Vert y\Vert _{E(\mathcal{M})^{(r_{1})}} \end{aligned}$$

for all $x\in E(\mathcal{M})^{(r_{0})}$ and $y\in E(\mathcal{M})^{(r _{1})}$ (see, inequality (1.3) in [p. 492, [13]]). Further details for commutative and noncommutative symmetric (quasi) Banach may be found in [11, 12, 14].

In what follows, *E* will always denote a minimal symmetric quasi-Banach space with order continuous norm.

## The joint log-convexity of two variables functionals relative to measurable operators

By slightly modifying the proof of Theorem 1 and Theorem 2 in [15], we get the following two lemmas.

### Lemma 3.1

*Let* $r>0$, $x, y\in \mathcal{M}^{+}$ *and* $z\in E(\mathcal{M})^{(r)}$. *Then*, *for* $0\leq s\leq 1$, *we have*

3.1 $$\begin{aligned} \bigl\Vert x^{s}zy^{s}\bigr\Vert _{E(\mathcal{M})^{(r)}}\leq \Vert z\Vert _{E(\mathcal{M})^{(r)}} ^{1-s}\Vert xzy \Vert _{E(\mathcal{M})^{(r)}}^{s}. \end{aligned}$$

### Proof

Let *I* be the set of all real numbers *s* in the interval $[0, 1]$ for which

$$\bigl\Vert x^{s}zy^{s}\bigr\Vert _{E(\mathcal{M})^{(r)}}\leq \Vert z\Vert _{E(\mathcal{M})^{(r)}} ^{1-s}\Vert xzy\Vert _{E(\mathcal{M})^{(r)}}^{s}. $$

By Lemma 2.5 of [5] and the triangle inequality, there exists a constant $C>0$ such that

$$\bigl\Vert x^{t}zy^{t}-x^{s}zy^{s} \bigr\Vert _{E(\mathcal{M})^{(r)}} \leq C \bigl(\bigl\Vert x^{t}-x^{s} \bigr\Vert \bigl\Vert y^{t}\bigr\Vert +\bigl\Vert y^{t}-y^{s} \bigr\Vert \bigl\Vert x^{s}\bigr\Vert \bigr)\Vert z\Vert _{E(\mathcal{M})^{(r)}} $$

for every $t, s> 0$. Based on this inequality, one can easily see that *I* is a closed subset of $[0, 1]$. Since $0, 1\in I$, the proof of the theorem will be complete if we show that *I* is convex, and so in this case $I=[0, 1]$. Suppose $t, s\in I$. We will show that $\frac{t+s}{2}\in I$.

For any $z\in E(\mathcal{M})^{(r)}$, there exists $u \in \mathcal{M}$ such that $z=u\vert z\vert $ is the polar decomposition of *z*. Put

$$z_{n}=u \vert z\vert e_{(\frac{1}{n}, n)} \bigl(\vert z\vert \bigr), $$

where $e_{(\frac{1}{n}, n)}(\vert z\vert )$ is the spectral projection of $\vert z\vert $ associated with the interval $(\frac{1}{n}, n)$. Since *E* is minimal, $\lim_{t\rightarrow \infty }\mu_{t}(\vert z\vert ^{r})=0$. According to [5, Proposition 3.2], we obtain $\tau (e_{(\frac{1}{n}, n)}(\vert z\vert ))< \infty $ for any $n\in \mathbb{N}$. Hence $\tau (e_{(\frac{1}{n}, n)}(\vert z\vert )) <\infty$, which guarantees that $z_{n}\in L^{1}( \mathcal{M})$. Combining Lemma 2.5(ii) and (iv) in [5] with [16, Lemma 2], we obtain

$$\begin{aligned} \bigl\Vert x^{\frac{t+s}{2}}z_{n}y^{\frac{t+s}{2}}\bigr\Vert _{E(\mathcal{M})^{(r)}} &= \bigl\Vert \mu \bigl(x^{\frac{t+s}{2}}z_{n}y^{\frac{t+s}{2}} \bigr)\bigr\Vert _{E^{(r)}} \\ &=\bigl\Vert \mu \bigl(y^{\frac{t+s}{2}}z_{n}^{*}x^{t+s}z_{n}y^{\frac{t+s}{2}} \bigr)^{\frac{1}{2}}\bigr\Vert _{E^{(r)}} \\ &=\bigl\Vert \mu \bigl(y^{t}z_{n}^{*}x^{t+s}z_{n}y^{s} \bigr)^{\frac{1}{2}}\bigr\Vert _{E^{(r)}} \\ &=\bigl\Vert \bigl(y^{t}z_{n}^{*}x^{t} \bigr) \bigl(x^{s}z_{n}y^{s} \bigr)\bigr\Vert _{E(\mathcal{M} )^{( \frac{r}{2})}}^{\frac{1}{2}} \\ &\leq \bigl\Vert y^{t}z_{n}^{*}x^{t} \bigr\Vert _{E(\mathcal{M})^{(r)}}^{\frac{1}{2}} \bigl\Vert x^{s}z_{n}y^{s} \bigr\Vert _{E(\mathcal{M})^{(r)}}^{\frac{1}{2}} \\ &\leq \Vert z_{n}\Vert ^{\frac{1-t}{2}}_{E(\mathcal{M})^{(r)}} \Vert xz_{n}y\Vert ^{\frac{t}{2}}_{E(\mathcal{M})^{(r)}} \Vert z_{n} \Vert ^{\frac{1-s}{2}}_{E( \mathcal{M})^{(r)}}\Vert xz_{n}y\Vert ^{\frac{s}{2}}_{E(\mathcal{M})^{(r)}} \\ &=\Vert z_{n}\Vert ^{1-\frac{t+s }{2}}_{E(\mathcal{M})^{(r)}}\Vert xz_{n}y\Vert ^{ \frac{t+s}{2}}_{E(\mathcal{M})^{(r)}}, \end{aligned}$$

thus $\frac{t+s }{2} \in I$. Therefore,

$$\bigl\Vert x^{s}z_{n}y^{s}\bigr\Vert _{E(\mathcal{M})^{(r)}}\leq \Vert z_{n}\Vert _{E( \mathcal{M})^{(r)}}^{1-s} \Vert xz_{n}y\Vert _{E(\mathcal{M})^{(r)}}^{s}. $$

Note that $\mu_{t}(z-z_{n})\leq \mu_{t}(ze_{[0, \frac{1}{n}]}(\vert z\vert )) \leq \frac{1}{n}\rightarrow 0$ and $\mu_{t}(z_{n})\geq \mu_{t}(z_{n+1})$, $t>0$. Since *E* has order continuous norm, we have

$$\Vert z-z_{n}\Vert _{E(\mathcal{M})^{(r)}}=\bigl\Vert ze_{[0, \frac{1}{n}]} \bigl(\vert z\vert \bigr)\bigr\Vert _{E(\mathcal{M})^{(r)}} \rightarrow 0. $$

On the other hand,

$$\begin{aligned} \bigl\Vert x^{s}zy^{s}-x^{s}z_{n}y^{s} \bigr\Vert _{E(\mathcal{M})^{(r)}} &=\bigl\Vert x^{s}(z-z_{n})y^{s} \bigr\Vert _{E(\mathcal{M})^{(r)}} \leq \bigl\Vert x^{s}\bigr\Vert \bigl\Vert y^{s} \bigr\Vert \Vert z-z_{n} \Vert _{E(\mathcal{M})^{(r)}}\rightarrow 0. \end{aligned}$$

This completes the proof. □

### Lemma 3.2

*Let* $r>0$, $x, y\in \mathcal{M}^{+}$ *and* $z\in E(\mathcal{M})^{(r)}$. *Then*, *for* $0\leq s\leq 1$, *we have*

3.2 $$\begin{aligned} \bigl\Vert x^{s}zy^{1-s}\bigr\Vert _{E(\mathcal{M})^{(r)}}\leq \Vert xz\Vert _{E(\mathcal{M})^{(r)}} ^{s} \Vert zy \Vert _{E(\mathcal{M})^{(r)}}^{1-s}. \end{aligned}$$

*In particular*, *if* $\mathcal{M}$ *is a finite von Neumann algebra*, *then* $\tau (x^{s}y^{1-s})\leq \tau (x)^{s}\tau (y)^{1-s}$.

### Proof

For each $\varepsilon >0$, let $y_{\varepsilon }=y+ \varepsilon 1$. Then $y_{\varepsilon }$ is invertible for every $\varepsilon >0$. Applying Lemma 3.1 to the operators *x*, $y_{\varepsilon }^{-1}$ and $zy_{\varepsilon }$, we get

$$\begin{aligned} \bigl\Vert x^{s}zy_{\varepsilon }^{1-s}\bigr\Vert _{E(\mathcal{M})^{(r)}} &=\bigl\Vert x^{s}(zy_{\varepsilon })y_{\varepsilon }^{-s} \bigr\Vert _{E(\mathcal{M})^{(r)}} \\ &\leq \Vert zy_{\varepsilon }\Vert _{E(\mathcal{M})^{(r)}}^{1-s} \bigl\Vert x(zy_{\varepsilon })y_{\varepsilon }^{-1}\bigr\Vert _{E(\mathcal{M})^{(r)}}^{s} \\ &\leq \Vert zy_{\varepsilon }\Vert _{E(\mathcal{M})^{(r)}}^{1-s} \Vert xz \Vert _{E( \mathcal{M})^{(r)}}^{s}. \end{aligned}$$

Letting $\varepsilon \rightarrow 0$ and using continuity arguments, we see that

$$\bigl\Vert x^{s}zy^{1-s}\bigr\Vert _{E(\mathcal{M})^{(r)}}\leq \Vert xz\Vert _{E(\mathcal{M})^{(r)}} ^{s}\Vert zy\Vert _{E(\mathcal{M})^{(r)}}^{1-s}. $$

 □

As in the proof of Lemma 3.2, it is easy to check that (3.1) and (3.2) are in fact equivalent.

In the following theorem we prove the joint log-convexity of the function $f(t, s)=\Vert x^{t}zy^{s}\Vert _{E(\mathcal{M})^{(r)}}$, which is one of our main results.

### Theorem 3.3

*Let* $r>0$ *and* *E* *be a symmetric quasi*-*Banach space*. *If* $x, y\in \mathcal{M}^{+}$ *and* $z\in E(\mathcal{M})^{(r)}$, *then* $f(t, s)=\Vert x^{t}zy^{s}\Vert _{E(\mathcal{M})^{(r)}}$ *is joint log*-*convex on* $(0, \infty)\times (0, \infty)$.

### Proof

First we assume that $x, y\in \mathcal{M}^{+}$ are invertible and $\alpha, \beta \geq 0$ with $\alpha +\beta =1$. Then Lemma 3.2 clearly implies the result. If either *x* or *y* is not invertible, a standard limit process will yield the result. □

### Remark 3.4

1. Let $r>0$ and *E* be a symmetric quasi-Banach space. If $x, y\in \mathcal{M}^{+}$ and $z\in E(\mathcal{M})^{(r)}$, then $f(t)=\Vert x^{t}zy^{1-t}\Vert _{E(\mathcal{M})^{(r)}}$ is log-convex on $[0, 1]$.
2. Let $r>0$ and *E* be a symmetric quasi-Banach space. If $x, y\in \mathcal{M}^{+}$ and $z\in E(\mathcal{M})^{(r)}$, then $f(s)=\Vert x^{1-s}zy^{s}\Vert _{E(\mathcal{M})^{(r)}}$ is log-convex on $[0, 1]$.
3. Let $r>0$ and *E* be a symmetric quasi-Banach space. If $x, y\in \mathcal{M}^{+}$ and $z\in E(\mathcal{M})^{(r)}$, then
  $$f(s)=\bigl\Vert x^{s}zy^{1-s}\bigr\Vert _{E(\mathcal{M})^{(r)}} \bigl\Vert x^{1-s}zy^{s}\bigr\Vert _{E( \mathcal{M})^{(r)}} $$
  is log-convex on $[0, 1]$. Moreover, $f(s)$ is decreasing on $[0, \frac{1}{2}]$ and increasing $[\frac{1}{2}, 1]$.

Next we present our second main result.

### Corollary 3.5

*Let* $r>0$ *and* *E* *be a symmetric quasi*-*Banach space*. *If* $x, y\in \mathcal{M}^{+}$ *and* $z\in E(\mathcal{M})^{(r)}$, *then*

$$f(s, t)=\bigl\Vert x^{\frac{t}{s}}zy^{\frac{t}{s}}\bigr\Vert _{E(\mathcal{M})^{(r)}} ^{s} =\bigl\Vert \bigl\vert x^{\frac{t}{s}}zy^{\frac{t}{s}} \bigr\vert ^{r}\bigr\Vert _{E(\mathcal{M})}^{ \frac{s}{r}} $$

*and*

$$F(s, t) =\bigl\Vert \bigl\vert x^{\frac{t}{s}}zy^{\frac{t}{s}} \bigr\vert ^{r}\bigr\Vert _{E(\mathcal{M})} ^{s} $$

*are joint log*-*convex on* $(0, \infty)\times (0, \infty)$.

### Proof

Applying Theorem 3.3 to $g(t)=\Vert x^{t}zy^{t} \Vert _{E(\mathcal{M})^{(r)}} $ indicates that $\log g(t)$ is convex on $(0, \infty)$. Thus the perspective *h* defined by

$$h(s, t)=t\log g \biggl(\frac{s}{t} \biggr)=\log g \biggl( \frac{s}{t} \biggr)^{t} $$

is a joint convex function in the sense that if $0\leq c\leq 1$, then

$$h \bigl(cs_{1}+(1-c)s_{2}, ct_{1}+(1-c)t_{2} \bigr)\leq ch(s_{1}, t_{1})+(1-c)h(s _{2}, t_{2}). $$

Therefore

$$f(s, t)=g \biggl(\frac{t}{s} \biggr)^{s} =\bigl\Vert x^{\frac{t}{s}}zy^{\frac{t}{s}}\bigr\Vert _{E( \mathcal{M})^{(r)}}^{s} = \bigl\Vert \bigl\vert x^{\frac{t}{s}}zy^{\frac{t}{s}} \bigr\vert ^{r}\bigr\Vert _{E(\mathcal{M})}^{\frac{s}{r}} $$

is joint log-convex on $(0, \infty)\times (0, \infty)$. Moreover,

$$f \bigl(\alpha (t_{1}, s_{1}) +(1-\alpha) (t_{2}, s_{2}) \bigr)^{r}\leq f(t_{1}, s _{1})^{r\alpha } f(t_{2}, s_{2})^{r(1-\alpha)}. $$

This implies that

$$F(s, t) =\bigl\Vert \bigl\vert x^{\frac{t}{s}}zy^{\frac{t}{s}} \bigr\vert ^{r}\bigr\Vert _{E(\mathcal{M})} ^{s} $$

is joint log-convex on $(0, \infty)\times (0, \infty)$. □

If $E=L_{2}$, in view of the log-convexity given in Theorem 3.3, we have the following trace inequalities.

### Proposition 3.6

*Let* $x, y\in \mathcal{M}^{+}$, $z\in L_{2}(\mathcal{M})$ *and* $p\geq q>0$. *Then*, *for* $r\leq q$, *we have*

$$\begin{aligned} \tau \bigl(x^{p}zy^{q}z^{*} \bigr) &\leq \bigl( \tau \bigl(x^{p+r}zy^{q-r}z^{*} \bigr) \bigr)^{\alpha } \bigl( \tau \bigl(x^{q-r}zy^{p+r}z^{*} \bigr) \bigr)^{\beta } \\ &\leq \alpha \tau \bigl(x^{p+r}zy^{q-r}z^{*} \bigr)+ \beta \tau \bigl(x^{q-r}zy^{p+r}z ^{*} \bigr), \end{aligned}$$

*where* $\alpha =\frac{p-q+r}{p-q+2r}$ *and* $\beta =1-\alpha$.

### Proof

The proof is immediate from Theorem 3.3. □

In what follows the *v*-version of the above inequalities is established.

### Corollary 3.7

*Let* $x, y\in \mathcal{M}^{+}$, $z\in L_{2}(\mathcal{M})$. *Then*, *for* $0\leq v\leq 1$, *we have*

$$\begin{aligned} \tau \bigl(x^{v}zy^{1-v}z^{*} \bigr) &\leq \bigl( \tau \bigl(x\vert z\vert ^{2} \bigr) \bigr)^{v} \bigl( \tau \bigl( y \bigl\vert z^{*} \bigr\vert ^{2} \bigr) \bigr)^{1-v} \\ &\leq v \tau \bigl(x\vert z\vert ^{2} \bigr)+(1-v)\tau \bigl( y \bigl\vert z^{*} \bigr\vert ^{2} \bigr). \end{aligned}$$

The following corollary deals with a log-convexity consequence, the matrix form of which is in itself a more generalized form when compared with the well-known Lieb’s concavity theorem and Ando’s convexity theorem. Moreover, it is very similar to Corollary 2.17 of [17]. Since the proof is very simple, the details are omitted.

### Corollary 3.8

*Let* $x, y\in \mathcal{M}^{+}$, $z\in L_{2}(\mathcal{M})$. *The function* $f: (0, \infty)\times (0, \infty)\rightarrow [0, \infty)$ *defined by* $f(p, q)=\tau (x^{p}zy^{q}z^{*})$ *is log*-*convex*.

One last remark about trace inequalities is the following monotonicity result. Simulating the method to prove Proposition 2.11 and Proposition 2.12 of [3], we can easily give its proof and we omit it.

### Corollary 3.9

*Let* $x, y\in \mathcal{M}^{+}$, $z\in L_{2}(\mathcal{M})$ *and* $0< q\leq p$. *The functions*

$$f(r)= \bigl(\tau \bigl(x^{p+r}zy^{q-r}z^{*} \bigr) \bigr)^{\frac{p-q+r}{p-q+2r}} \bigl(\tau \bigl(x ^{q-r}zy^{p+-r}z^{*} \bigr) \bigr)^{\frac{r}{p-q+2r}} $$

*and*

$$f(r)=\frac{p-q+r}{p-q+2r}\tau \bigl(x^{p+r}zy^{q-r}z^{*} \bigr) + \frac{r}{p-q+2r} \tau \bigl(x^{q-r}zy^{p+-r}z^{*} \bigr) $$

*are increasing on* $[0, q]$.

In the following we will give another main result, which is a generalization of [1, Theorem 1.2]. To establish the result, we need the following lemma.

### Lemma 3.10

*Let* $r>0$ *and* *E* *be a symmetric quasi*-*Banach space*. *If* $x, y\in \mathcal{M}^{+}$ *and* $z\in E(\mathcal{M})^{(r)}$, *then for all* $\alpha >0$, $f(t)=\Vert \vert x^{t}zy^{t}\vert ^{\alpha }\Vert _{E(\mathcal{M})^{(r)}}$ *is log*-*convex on* $(0,\infty)$.

### Proof

By a similar argument to that in the proof of Theorem 3.3, it suffices to prove the case when $x, y\in \mathcal{M}^{+}$ are invertible. Let $x, y\in \mathcal{M}^{+}$ be invertible and $\beta, \gamma \geq 0$ with $\beta +\gamma =1$. According to Lemma 3.2, we get

$$\begin{aligned} f(\beta t+\gamma s) &=\bigl\Vert \bigl\vert x^{\beta t+\gamma s}zy^{\beta t+\gamma s} \bigr\vert ^{\alpha }\bigr\Vert _{E(\mathcal{M})^{(r)}} \\ &=\bigl\Vert \bigl\vert x^{(t-s)\beta }x^{s}zy^{t}y^{(s-t)\gamma } \bigr\vert ^{\alpha }\bigr\Vert _{E( \mathcal{M})^{(r)}} \\ &\leq \bigl\Vert \bigl\vert x^{t}zy^{t} \bigr\vert ^{\alpha }\bigr\Vert _{E(\mathcal{M})^{(r)}}^{\beta } \cdot \bigl\Vert \bigl\vert x^{s}zy^{s} \bigr\vert ^{\alpha }\bigr\Vert _{E(\mathcal{M})^{(r)}}^{\gamma }. \end{aligned}$$

 □

Now we are in the position to give the main result.

### Theorem 3.11

*Let* $r>0$ *and* *E* *be a symmetric quasi*-*Banach space*. *If* $x, y\in \mathcal{M}^{+}$ *and* $z\in E(\mathcal{M})^{(r)}$, *then for all* $\alpha >0$, $f(p,t)=\Vert \vert x^{\frac{t}{p}}zy^{\frac{t}{p}}\vert ^{\alpha p}\Vert _{E(\mathcal{M})^{(r)}}$ *is joint log*-*convex on* $(0,\infty)\times (0, \infty)$.

### Proof

Let $x, y\in \mathcal{M}^{+}$ and $\beta, \gamma \geq 0$ with $\beta +\gamma =1$. Firstly we have

$$\begin{aligned} f(p,t) &=\bigl\Vert \bigl\vert x^{\frac{t}{p}}zy^{\frac{t}{p}} \bigr\vert ^{\alpha p}\bigr\Vert _{E( \mathcal{M})^{(r)}} =\bigl\Vert \bigl\vert x^{\frac{t}{p}}zy^{\frac{t}{p}} \bigr\vert ^{\alpha p\cdot r}\bigr\Vert _{E( \mathcal{M})}^{\frac{1}{r}} =\bigl\Vert \bigl\vert x^{\frac{t}{p}}zy^{\frac{t}{p}} \bigr\vert ^{\alpha }\bigr\Vert _{E(\mathcal{M})^{(pr)}} ^{p}. \end{aligned}$$

Write $g(t)=\Vert \vert x^{t}zy^{t}\vert ^{\alpha }\Vert _{E(\mathcal{M})^{(pr)}}$. It follows from Lemma 3.10 that $g(t)$ is log-convex on $(0,\infty)$. As in the proof of Corollary 3.5, we show that

$$f(p,t)=g \biggl(\frac{t}{p} \biggr)^{p}=\bigl\Vert \bigl\vert x^{\frac{t}{p}}zy^{\frac{t}{p}} \bigr\vert ^{\alpha}\bigr\Vert _{E(\mathcal{M})^{(pr)}}^{p} $$

is joint log-convex on $(0,\infty)\times (0,\infty)$. For the case either *x* or *y* is not invertible, a standard limit process yields the result. □

The novelty of the proofs of Corollary 3.5 and Theorem 3.11 is applying the perspective of a one variable convex function of measurable operators. See [18] for more information and details on noncommutative perspectives. Moreover, one more perspective yields the following result, which is also a special case of Theorem 3.11.

### Corollary 3.12

*Let* $r>0$ *and* *E* *be a symmetric quasi*-*Banach space*. *If* $x, y\in \mathcal{M}^{+}$ *and* $z\in E(\mathcal{M})^{(r)}$, *then for all* $\alpha >0$, $f(p)=\Vert \vert x^{\frac{1}{p}}zy^{\frac{1}{p}}\vert ^{\alpha }\Vert _{E(\mathcal{M})^{(r)}}^{p}$ *is log*-*convex on* $(0, \infty)$.

### Proof

Let $p=1$ in Theorem 3.11, we see that $\Phi (t)=\log (\Vert \vert x^{t}zy^{t}\vert ^{\alpha }\Vert _{E(\mathcal{M})^{(r)}})$ is convex on $(0, \infty)$, hence its perspective

$$p\Phi \biggl(\frac{t}{p} \biggr)=\log \bigl(\bigl\Vert \bigl\vert x^{\frac{t}{p}}zy^{\frac{t}{p}} \bigr\vert ^{\alpha}\bigr\Vert _{E(\mathcal{M})^{(r)}}^{p} \bigr) $$

is joint convex on $(0,\infty)\times (0,\infty)$. The proof is to be completed by fixing $t=1$. □

Next we state a result related to Theorem 3.11; since the proof is immediate from Theorem 3.11, we omit it.

### Corollary 3.13

*Let* $r>0$ *and* *E* *be a symmetric quasi*-*Banach space*. *If* $x\in \mathcal{M}^{+}$ *and* $z\in E(\mathcal{M})^{(r)}$, *then for all* $\alpha >0$, $f(p,t)=\Vert \vert z^{*}x^{\frac{t}{p}}z\vert ^{\alpha p}\Vert _{E( \mathcal{M})^{(r)}}$ *is joint log*-*convex on* $(0,\infty)\times (0, \infty)$.

## Interpolated Young and Heinz inequalities and refined forms

As another application of the log-convexity shown in Theorem 3.3, we obtain the following interpolated Young inequalities.

### Theorem 4.1

*Let* *E* *be a symmetric quasi*-*Banach space and* $x, y\in \mathcal{M} ^{+}$ *and* $z\in E(\mathcal{M})$. *Then*, *for* $0\leq r\leq q\leq p$, *we have*

4.1 $$\begin{aligned} \bigl\Vert x^{p}zy^{q}\bigr\Vert _{E(\mathcal{M})}\leq \bigl\Vert x^{p+r}zy^{q-r}\bigr\Vert _{E( \mathcal{M})}^{\alpha }\bigl\Vert x^{q-r}zy^{p+r}\bigr\Vert _{E(\mathcal{M})}^{\beta } \end{aligned}$$

*and*

4.2 $$\begin{aligned} \bigl\Vert x^{p}zy^{q}\bigr\Vert _{E(\mathcal{M})}\leq \alpha \bigl\Vert x^{p+r}zy^{q-r}\bigr\Vert _{E( \mathcal{M})}+ \beta \bigl\Vert x^{q-r}zy^{p+r}\bigr\Vert _{E(\mathcal{M})}, \end{aligned}$$

*where*

$$\alpha =\frac{p-q+r}{p-q+2r},\qquad \beta =\frac{r}{p-q+2r}. $$

### Proof

The proof can be done similarly to that of Theorem 2.2 in [3]. □

### Lemma 4.2

*Let* $r>0$ *and* *E* *be a symmetric quasi*-*Banach space*. *If* $x, y\in \mathcal{M}^{+}$ *and* $z\in E(\mathcal{M})^{(r)}$, *then for* $0\leq p \leq t\leq s$, *we have*

$$\bigl\Vert x^{s}zy^{t}\bigr\Vert _{E(\mathcal{M})^{(r)}}\leq \bigl\Vert x^{s+p}zy^{t-p}\bigr\Vert _{E( \mathcal{M})^{(r)}}^{\alpha } \bigl\Vert x^{t-p}zy^{s+p}\bigr\Vert _{E(\mathcal{M})^{(r)}} ^{\beta }, $$

*where* $\alpha =\frac{s-t+p}{s-t+2p}$, $\beta =\frac{p}{s-t+2p}$.

### Proof

Applying Theorem 3.3 to $f(s, t)= \Vert x^{s}zy^{t}\Vert _{E(\mathcal{M})^{(r)}}$ yields the desired result. □

In what follows, we turn our attention to more refined interpolated inequalities. Moreover, unless stated otherwise, in the sequel, *α* and *β* will refer to those of Lemma 4.2.

### Theorem 4.3

*Let* $r>0$ *and* *E* *be a symmetric quasi*-*Banach space*. *If* $x, y\in \mathcal{M}^{+}$ *and* $z\in E(\mathcal{M})^{(r)}$, *then for* $0\leq p \leq t\leq s$, *we have*

$$\begin{aligned} &\bigl\Vert x^{s}zy^{t}\bigr\Vert _{E(\mathcal{M})^{(r)}}^{2}+ \beta^{2} \bigl(\bigl\Vert x^{s+p}zy^{t-p}\bigr\Vert _{E(\mathcal{M})^{(r)}}- \bigl\Vert x^{t-p}zy^{s+p}\bigr\Vert _{E(\mathcal{M})^{(r)}} \bigr)^{2} \\ &\quad \leq \bigl(\alpha \bigl\Vert x^{s+p}zy^{t-p}\bigr\Vert _{E(\mathcal{M})^{(r)}}+\beta \bigl\Vert x^{t-p}zy^{s+p}\bigr\Vert _{E(\mathcal{M})^{(r)}} \bigr)^{2}. \end{aligned}$$

### Proof

The theorem can be easily proved by using Lemma 4.2 and the method to prove Theorem 2.3 of [3]. □

It is worth to remark that a *v*-version of the above refined interpolated inequality has not been given in the literature, and we present it here without a proof since it is immediate from Theorem 4.3.

### Corollary 4.4

*Let* $r>0$ *and* *E* *be a symmetric quasi*-*Banach space*. *If* $x, y\in \mathcal{M}^{+}$ *and* $z\in E(\mathcal{M})^{(r)}$, *then for* $0\leq v \leq 1$, *we have*

$$\begin{aligned} &\bigl\Vert x^{v}zy^{1-v}\bigr\Vert _{E(\mathcal{M})^{(r)}}^{2}+r_{0}^{2} \bigl( \Vert xz\Vert _{E( \mathcal{M})^{(r)}}-\Vert zy\Vert _{E(\mathcal{M})^{(r)}} \bigr)^{2} \\ &\quad \leq \bigl(v\Vert xz\Vert _{E(\mathcal{M})^{(r)}}+(1-v)\Vert zy\Vert _{E(\mathcal{M})^{(r)}} \bigr)^{2}, \end{aligned}$$

*where* $r_{0}=\min \{v,1-v\}$.

One may compare this inequality with the one given in Theorem 3.8 of [19]. And in just the same way as in [3, Theorem 2.5], we establish the following interpolation inequality.

### Proposition 4.5

*Let* $r>0$ *and* *E* *be a symmetric quasi*-*Banach space*. *If* $x, y\in \mathcal{M}^{+}$ *and* $z\in E(\mathcal{M})^{(r)}$, *then for* $0\leq p \leq t\leq s$, *we have*

$$\begin{aligned} &\bigl\Vert x^{s}zy^{t}\bigr\Vert _{E(\mathcal{M})^{(r)}}+ \beta \bigl(\bigl\Vert x^{s+p}zy^{t-p}\bigr\Vert _{E(\mathcal{M})^{(r)}}^{\frac{1}{2}}- \bigl\Vert x^{t-p}zy^{s+p} \bigr\Vert _{E( \mathcal{M})^{(r)}}^{\frac{1}{2}} \bigr)^{2} \\ &\quad \leq \alpha \bigl\Vert x^{s+p}zy^{t-p}\bigr\Vert _{E(\mathcal{M})^{(r)}}+\beta \bigl\Vert x^{t-p}zy^{s+p}\bigr\Vert _{E(\mathcal{M})^{(r)}}. \end{aligned}$$

Next we present a different refined interpolated inequality. Additionally, this version was first given for matrices in [3]. Since the proof is very simple by an application of Lemma 4.2 and a straightforward calculation, the details are omitted.

### Proposition 4.6

*Let* $r>0$ *and* *E* *be a symmetric quasi*-*Banach space*. *If* $x, y\in \mathcal{M}^{+}$ *and* $z\in E(\mathcal{M})^{(r)}$, *then for* $0\leq p \leq t\leq s$, *we have*

$$\begin{aligned} &(\alpha -\beta)^{2\beta }\bigl\Vert x^{s}zy^{t} \bigr\Vert _{E(\mathcal{M})^{(r)}}^{2} +\beta^{2} \bigl(\bigl\Vert x^{s+p}zy^{t-p}\bigr\Vert _{E(\mathcal{M})^{(r)}}+\bigl\Vert x^{t-p}zy^{s+p}\bigr\Vert _{E(\mathcal{M})^{(r)}} \bigr)^{2} \\ &\quad \leq \bigl(\alpha \bigl\Vert x^{s+p}zy^{t-p}\bigr\Vert _{E(\mathcal{M})^{(r)}}+\beta \bigl\Vert x^{t-p}zy^{s+p}\bigr\Vert _{E(\mathcal{M})^{(r)}} \bigr)^{2}. \end{aligned}$$

Analogously, we give now a *v*-version of the above inequality.

### Corollary 4.7

*Let* $r>0$ *and* *E* *be a symmetric quasi*-*Banach space*. *If* $x, y\in \mathcal{M}^{+}$ *and* $z\in E(\mathcal{M})^{(r)}$, *then for* $0\leq v \leq 1$, *we have*

$$\begin{aligned} &\vert 1-2v\vert ^{2r_{0}}\bigl\Vert x^{v}zy^{1-v} \bigr\Vert _{E(\mathcal{M})^{(r)}}^{2}+r_{0} ^{2} \bigl( \Vert xz\Vert _{E(\mathcal{M})^{(r)}}+\Vert zy\Vert _{E(\mathcal{M})^{(r)}} \bigr)^{2} \\ &\quad \leq \bigl(v\Vert xz\Vert _{E(\mathcal{M})^{(r)}}+(1-v)\Vert zy\Vert _{E(\mathcal{M})^{(r)}} \bigr)^{2}, \end{aligned}$$

*where* $r_{0}=\min \{v,1-v\}$.

The following inequality is a refined interpolated Heinz inequality.

### Theorem 4.8

*Let* $r>0$ *and* *E* *be a symmetric quasi*-*Banach space*. *If* $x, y\in \mathcal{M}^{+}$ *and* $z\in E(\mathcal{M})^{(r)}$, *then for* $0\leq p \leq t\leq s$, *we have*

$$\begin{aligned} &\bigl\Vert x^{s}zy^{t}\pm x^{t}zy^{s} \bigr\Vert _{E(\mathcal{M})^{(r)}}^{2} +2\beta \bigl( \bigl\Vert x^{s+p}zy^{t-p}\bigr\Vert _{E(\mathcal{M})^{(r)}}^{\frac{1}{2}}- \bigl\Vert x^{t-p}zy^{s+p}\bigr\Vert _{E(\mathcal{M})^{(r)}}^{\frac{1}{2}} \bigr)^{2} \\ &\quad \leq \bigl(\bigl\Vert x^{s+p}zy^{t-p}\bigr\Vert _{E(\mathcal{M})^{(r)}}+\bigl\Vert x^{t-p}zy^{s+p}\bigr\Vert _{E(\mathcal{M})^{(r)}} \bigr)^{2}. \end{aligned}$$

### Proof

Lemma 4.2 and Corollary 2.2 of [20] yield the conclusion. □

### Remark 4.9

Note that the above inequality should be compared with

4.3 $$\begin{aligned} \bigl\Vert x^{v}zy^{1-v}+x^{1-v}zy^{v} \bigr\Vert _{p}+2r_{0} \bigl(\Vert xz\Vert _{p}^{\frac{1}{2}}- \Vert zy\Vert _{p}^{\frac{1}{2}} \bigr)^{2}\leq \Vert xz\Vert _{p}+\Vert zy \Vert _{p}, \end{aligned}$$

where $0\leq v\leq 1$ and $r_{0}=\min \{v,1-v\}$, proved in [19], when we particularly take the case of $E=L_{p}$ into consideration.

In addition, if we take $v=\frac{1}{2}$ in (4.3), we get

4.4 $$\begin{aligned} 2\bigl\Vert x^{\frac{1}{2}}zy^{\frac{1}{2}}\bigr\Vert _{p}+ \bigl(\Vert xz\Vert _{p}^{\frac{1}{2}}- \Vert zy \Vert _{p}^{\frac{1}{2}} \bigr)^{2}\leq \Vert xz\Vert _{p}+\Vert zy\Vert _{p}. \end{aligned}$$

The following result is a related interpolated inequality. The proof is immediate by using Lemma 4.2 and we omit it.

### Proposition 4.10

*Let* $r>0$ *and* *E* *be a symmetric quasi*-*Banach space*. *If* $x, y\in \mathcal{M}^{+}$ *and* $z\in E(\mathcal{M})^{(r)}$, *then for* $0\leq p \leq t\leq s$, *we have*

$$\begin{aligned} &2\bigl\Vert x^{s}zy^{t}\bigr\Vert _{E(\mathcal{M})^{(r)}}^{\frac{1}{2}} + \bigl(\bigl\Vert x^{s+p}zy^{t-p} \bigr\Vert _{E(\mathcal{M})^{(r)}}^{\frac{\alpha }{2}}- \bigl\Vert x^{t-p}zy^{s+p} \bigr\Vert _{E(\mathcal{M})^{(r)}}^{\frac{\beta }{2}} \bigr)^{2} \\ &\quad \leq \bigl\Vert x^{s+p}zy^{t-p}\bigr\Vert _{E(\mathcal{M})^{(r)}}^{\alpha }+ \bigl\Vert x^{t-p}zy^{s+p} \bigr\Vert _{E(\mathcal{M})^{(r)}}^{\beta }. \end{aligned}$$

Observe that, if $s=t=p=\frac{1}{2}$, the above inequality is

$$2\bigl\Vert x^{\frac{1}{2}}zy^{\frac{1}{2}}\bigr\Vert _{E(\mathcal{M})^{(r)}}^{ \frac{1}{2}} + \bigl(\Vert xz\Vert _{E(\mathcal{M})^{(r)}}^{\frac{1}{4}}-\Vert zy\Vert _{E( \mathcal{M})^{(r)}}^{\frac{1}{4}} \bigr)^{2}\leq \Vert xz\Vert _{E(\mathcal{M})^{(r)}} ^{\frac{1}{2}}+\Vert zy\Vert _{E(\mathcal{M})^{(r)}}^{\frac{1}{2}}. $$

This inequality is equivalent to (4.4). This can be seen by expanding and using the fact that $\Vert x^{\frac{1}{2}}zy^{\frac{1}{2}}\Vert _{E(\mathcal{M})^{(r)}}\leq \Vert xz\Vert _{E(\mathcal{M})^{(r)}}^{ \frac{1}{2}}\cdot \Vert zy\Vert _{E(\mathcal{M})^{(r)}}^{\frac{1}{2}}$ (Corollary 3.4 of [21]).

Next we present the monotonicity of the interpolating terms in Theorem 4.1.

### Proposition 4.11

*Let* $r>0$ *and* *E* *be a symmetric quasi*-*Banach space*. *If* $x, y\in \mathcal{M}^{+}$ *and* $z\in E(\mathcal{M})^{(r)}$, *then for* $0< t\leq s$, *we have*

$$f(p)=\frac{s-t+p}{s-t+2p} \bigl\Vert x^{s+p}zy^{t-p}\bigr\Vert _{E(\mathcal{M})^{(r)}}+ \frac{p}{s-t+2p} \bigl\Vert x^{t-p}zy^{s+p} \bigr\Vert _{E(\mathcal{M})^{(r)}} $$

*is increasing on* $[0, t]$.

### Proof

Let $0< p_{1}, p_{2}\leq t$. It follows from Lemma 4.2 and the usual Young inequality that

$$\bigl\Vert x^{s}zy^{t}\bigr\Vert _{E(\mathcal{M})^{(r)}}\leq \frac{s-t+p_{1}}{s-t+2p _{1}} \bigl\Vert x^{s+p_{1}}zy^{t-p_{1}}\bigr\Vert _{E(\mathcal{M})^{(r)}} + \frac{p _{1}}{s-t+2p_{1}} \bigl\Vert x^{t-p_{1}}zy^{s+p_{1}} \bigr\Vert _{E(\mathcal{M})^{(r)}}. $$

Taking $p=p_{2}-p_{1}$ in Lemma 4.2 and applying the usual Young inequality to $\Vert x^{s+p_{1}}z y^{t-p_{1}}\Vert _{E(\mathcal{M})^{(r)}}$, we get

$$\begin{aligned} &\bigl\Vert x^{s+p_{1}}zy^{t-p_{1}}\bigr\Vert _{E(\mathcal{M})^{(r)}} \\ &\quad \leq \frac{(s+p_{1})-(t-p_{1})+(p_{2}-p_{1})}{(s+p_{1})-(t-p_{1})+2(p _{2}-p_{1})} \bigl\Vert x^{s+p_{1}+(p_{2}-p_{1})}zy^{t-p_{1}-(p_{2}-p_{1})}\bigr\Vert _{E(\mathcal{M})^{(r)}} \\ &\qquad {}+ \frac{p_{2}-p_{1}}{(s+p_{1})-(t-p_{1})+2(p_{2}-p_{1})} \bigl\Vert x^{t-p_{1}-(p_{2}-p_{1})}zy^{s+p_{1}+(p_{2}-p_{1})}\bigr\Vert _{E(\mathcal{M})^{(r)}} \\ &\quad =\frac{s-t+p_{1}+p_{2}}{s-t+2p_{1}} \bigl\Vert x^{s+p_{2}}zy^{t-p_{2}}\bigr\Vert _{E( \mathcal{M})^{(r)}} +\frac{p_{1}+p_{2}}{s-t+2p_{1}} \bigl\Vert x^{t-p_{2}}zy^{s+p_{2}} \bigr\Vert _{E(\mathcal{M})^{(r)}}. \end{aligned}$$

Similarly,

$$\begin{aligned} \bigl\Vert x^{t-p_{1}}zy^{s+p_{1}}\bigr\Vert _{E(\mathcal{M})^{(r)}} \leq& \frac{ p_{2}-p _{1}}{s-t+2p_{1}} \bigl\Vert x^{s+p_{2}}zy^{t-p_{2}}\bigr\Vert _{E(\mathcal{M})^{(r)}} \\ &{}+\frac{s-t+p_{1}+p_{2}}{s-t+2p_{1}} \bigl\Vert x^{t-p_{2}}zy^{s+p_{2}}\bigr\Vert _{E( \mathcal{M})^{(r)}}. \end{aligned}$$

As in the proof of the result in [3], the above two inequalities yield the desired consequence. □

This monotonicity tells us that $f(0)\leq f(t)$, i.e.,

4.5 $$\begin{aligned} \bigl\Vert x^{s}zy^{t}\bigr\Vert _{E(\mathcal{M})^{(r)}}\leq \frac{s}{s+t}\bigl\Vert x^{s+t}z\bigr\Vert _{E(\mathcal{M})^{(r)}}+\frac{t}{s+t}\bigl\Vert zy^{s+t}\bigr\Vert _{E(\mathcal{M})^{(r)}}, \end{aligned}$$

where $r>0$, $x, y\in \mathcal{M}^{+}$ and $z\in E(\mathcal{M})^{(r)}$. Note that inequality (4.5) is a noncommutative analogue of the $(s, t)$-version of Young inequality:

$$a^{s}b^{t}\leq \frac{s}{s+t}a^{s+t}+ \frac{t}{s+t}b^{s+t}, \quad a, b, s, t>0. $$

Moreover, based on Proposition 4.11, we derive the following result.

### Proposition 4.12

*Let* $r>0$ *and* *E* *be a symmetric quasi*-*Banach space*. *If* $x, y\in \mathcal{M}^{+}$ *and* $z\in E(\mathcal{M})^{(r)}$, *then for* $0< t\leq s$, *we have*

$$f(p)= \bigl\Vert x^{s+p}zy^{t-p}\bigr\Vert _{E(\mathcal{M})^{(r)}}^{ \frac{s-t+p}{s-t+2p}} \cdot \bigl\Vert x^{t-p}zy^{s+p} \bigr\Vert _{E(\mathcal{M})^{(r)}} ^{\frac{p}{s-t+2p}} $$

*is increasing on* $[0, t]$.

### Proof

Let $0\leq p_{1}< p_{2}\leq t$ and put $\alpha_{1}=\frac{s-t+p _{1}}{s-t+2p_{1}}$, $\beta_{1}=1-\alpha_{1}$, $\alpha_{2}=\frac{s-t+p_{1}+p _{2}}{s-t+2p_{1}}$, $\beta_{2}=1-\alpha_{2}$. Thanks to Lemma 4.2 and the computations of Proposition 4.11, we can show the assertion. □

### Remark 4.13

For $0\leq v\leq 1$, write $t=\min \{v, 1-v\}$, $s=\max \{v, 1-v\}$, from Proposition 4.12, it follows that

$$\bigl\Vert x^{s}zy^{t}\bigr\Vert _{E(\mathcal{M})^{(r)}}\leq \Vert xz\Vert _{E(\mathcal{M})^{(r)}} ^{s}\cdot \Vert zy\Vert _{E(\mathcal{M})^{(r)}}^{t}, $$

which is a generalization of Theorem 3 of [22].

## The Heinz means and its monotonicity

In this section, let $\mathcal{M}$ be a finite von Neumann algebra acting on the Hilbert space $\mathcal{H}$ with a normal finite faithful trace *τ*. Recall that if $x, y\in L_{2}(\mathcal{M})$ are positive and $z\in \mathcal{M}$, then the function $f(v)=\Vert x^{v}zy^{1-v}+x^{1-v}zy^{v}\Vert _{2}$ is convex on $[0,1]$ (see [19] for details). In what follows, we will first prove the convexity of $f(v)$ on $\mathbb{R}$.

### Theorem 5.1

*Let* $x, y\in L_{2}(\mathcal{M})^{+}$ *be invertible*, *and let* $z\in \mathcal{M}$, *then the function*

$$f(v)=\bigl\Vert x^{v}zy^{1-v}+x^{1-v}zy^{v} \bigr\Vert _{2} $$

*is convex on* $\mathbb{R}$.

### Proof

Using the proof method of Lemma 3.3 of [19] and Theorem 3.18 of [23], we can easily get the conclusion. □

Having proved the convexity of $f(v)=\Vert x^{v}zy^{1-v}+x^{1-v}zy^{v}\Vert _{2}$ on $\mathbb{R}$, the consequence which follows is an immediate result of Theorem 2.2 of [23], which is in fact the reversed version of the Heinz means inequality of *τ*-measurable operators in Corollary 3.5 in [19]:

$$\Vert xz+zy\Vert _{2}+2v \bigl(\Vert xz+zy\Vert _{2}-2\bigl\Vert x^{\frac{1}{2}}zy^{\frac{1}{2}}\bigr\Vert _{2} \bigr)\leq \bigl\Vert x^{-v}zy^{1+v}+x^{1+v}zy^{-v} \bigr\Vert _{2} $$

for $x, y\in L_{2}(\mathcal{M})$, $z\in \mathcal{M}$, $0\leq v\leq 1$.

In what follows, we try to describe the Heinz means of *τ*-measurable operators for $v\notin [0,1]$.

### Proposition 5.2

*Let* $x, y\in L_{2}(\mathcal{M})^{+}$ *be invertible and* $z\in \mathcal{M}$, *then*

$$\Vert xz+zy\Vert _{2}\leq \bigl\Vert x^{v}zy^{1-v}+x^{1-v}zy^{v} \bigr\Vert _{2}, \quad v\notin [0,1]. $$

### Proof

For $v\notin [0,1]$, the conclusion is immediate with *v* replaced by $v/{2v-1}$ and *x*, *y*, *z* by $x^{2v-1}$, $y^{2v-1}$, $x^{1-v}zy^{1-v}$, respectively, in Corollary 3.5 of [19]. □

### Proposition 5.3

*Let* $x, y\in L_{2}(\mathcal{M})^{+}$ *be invertible*, $z\in \mathcal{M}$ *and* $0< q< p$. *Then*

$$\bigl\Vert x^{p}zy^{-q}+x^{-q}zy^{p} \bigr\Vert _{2}\geq \bigl\Vert x^{p-q}z+zy^{p-q} \bigr\Vert _{2}. $$

### Proof

For such *x*, *y*, *p*, *q*, let $w=x^{p-q}$, $u=y^{p-q}$, $v=\frac{p}{p-q}$, thus $v>1$ and the consequence follows by a straightforward calculation with Proposition 5.2. □

Next we give an interpolated version, which will help prove the monotonicity of the Heinz means for $v\in \mathbb{R}$.

### Proposition 5.4

*Let* $x, y\in L_{2}(\mathcal{M})^{+}$ *be invertible*, $z\in \mathcal{M}$ *and* $0< r< q< p$. *Then*

$$\bigl\Vert x^{p}zy^{-q}+x^{-q}zy^{p} \bigr\Vert _{2}\geq \bigl\Vert x^{p-r}zy^{-q+r}+x^{-q+r}zy^{p-r} \bigr\Vert _{2}. $$

### Proof

Note that the assumption $0< r< q< p$ indicates $p+q-r>r$. Now the result follows from Proposition 5.3, with *p*, *q* replaced by $p+q-r$, *r* and with *z* replaced by $x^{-q+r}zy ^{-q+r}$. □

To proceed further, we only need to prove the following monotonicity result for the interpolated version.

### Proposition 5.5

*Let* $x, y\in L_{2}(\mathcal{M})^{+}$ *be invertible*, $z\in \mathcal{M}$ *and* $0< q< p$. *Then the function*

$$f(r)=\bigl\Vert x^{p-r}zy^{-q+r}+x^{-q+r}zy^{p-r} \bigr\Vert _{2} $$

*is decreasing on* $[0,q]$.

### Proof

Let $0\leq r_{1}\leq r_{2}\leq q$. Thus the result follows immediately by using Proposition 5.4 with *p*, *q*, *r* respectively replaced by $p-r_{1}$, $-q+r_{1}$, $r_{2}-r _{1}$. □

Now we are ready for the monotonicity of the Heinz means.

### Theorem 5.6

*Let* $x, y\in L_{2}(\mathcal{M})^{+}$ *be invertible and* $z\in \mathcal{M}$, *let* $f(v)=\Vert x^{v}zy^{1-v}+x^{1-v}zy^{v}\Vert _{2}$, *then* *f* *is decreasing on* $(-\infty, \frac{1}{2}]$ *and is increasing on* $[\frac{1}{2}, +\infty)$.

### Proof

The proof can be done similarly to that of Theorem 3.23 in [23]. □

### Remark 5.7

1. Notice that Proposition 5.4 is immediate from Proposition 5.5.
2. Using the monotonicity studied in Theorem 5.6, we have $f(\frac{1}{2})\leq f(0)$, i.e.,
  $$2\bigl\Vert x^{\frac{1}{2}}zy^{\frac{1}{2}}\bigr\Vert _{2}\leq \Vert xz+zy\Vert _{2}, $$
  which is obviously the Young inequality for the norm $\Vert \cdot \Vert _{2}$. Moreover, by a direct application of Theorem 5.6, we derive Proposition 5.2.
